# Managing Seasonal Infertility in Sows: Parity and Farm-Specific In-Barn Environmental Predictors of Reproductive Performance

**DOI:** 10.3390/vetsci13070607

**Published:** 2026-06-24

**Authors:** Isabela Cristina Colaço Bez, Ana Julia Carrasco Buzatto, Claudia Sevillano, Marcos Lopes, Saulo Henrique Weber, Leandro Batista Costa

**Affiliations:** 1 MonoHub—Research Group for Monogastric Animals, Graduate Program of Animal Science, School of Medicine and Life Sciences, Pontifícia Universidade Católica do Paraná, Imaculada Conceição Street, 1155, Curitiba 80215-901, Brazil; isabela.bez@pucpr.edu.br; 2 Graduate Program of Animal Science, School of Medicine and Life Sciences, Pontifícia Universidade Católica do Paraná, Imaculada Conceição Street, 1155, Curitiba 80215-901, Brazilsaulo.weber@pucpr.br (S.H.W.); 3 Topigs Norsvin Research Center B.V., P.O. Box 43, 6640 AA Beuningen, The Netherlands; 4 Topigs Norsvin, Visconde do Rio Branco Street, 1310, Curitiba 80420-210, Brazil

**Keywords:** farrowing success, farm management, heat load, litter size, photoperiod, pig

## Abstract

Seasonal infertility remains a major challenge in pig production and is influenced by environmental conditions and sow parity. This study aimed to identify in-barn heat load (HL), light variability (LV), and parity as farm-specific predictors of farrowing success (FS) and litter size (LS) in purebred Large White sows raised under subtropical and tropical conditions in Brazil. Reproductive records from 2021 were obtained from two commercial farms, and in-barn temperature and illuminance were recorded using data loggers installed in gestation units. Services between March and August 2021 were analyzed using logistic regression for FS and linear regression for LS, resulting in 732 service records from Farm 1 and 233 from Farm 2. In the combined analysis, parity was associated with higher odds of FS (OR = 1.20; *p* = 0.003) and greater LS (β = 0.47; *p* < 0.001). HL was not associated with FS but showed a small positive association with LS (β = 0.28; *p* = 0.031). In Farm 2, LV was negatively associated with FS (OR = 0.72; *p* = 0.028) and LS (β = −0.59; *p* = 0.020). Overall, parity structure strongly shaped reproductive performance, and in-barn environmental effects were context-dependent.

## Simple Summary

Seasonal infertility in sows affects reproductive performance, primarily by reducing farrowing rates and litter sizes. This study examined whether barn temperature, daily light exposure variability, and sow parity could predict the effect of these environmental factors in these reproductive outcomes. Data were collected from two commercial pig farms in Brazil, one in a subtropical region and one in a tropical region, during the period most commonly associated with seasonal reproductive decline. The results showed that sows with higher parity were consistently more likely to farrow successfully and produce larger litters, making the age and reproductive experience of the herd the strongest predictor of performance. The effect of temperature and lighting, however, varied between farms. On the tropical farm, greater day-to-day instability in barn lighting was associated with fewer successful farrowings and smaller litters, suggesting that inconsistent light exposure may be an underappreciated stressor. These findings highlight the importance of tailoring management strategies to each farm’s specific conditions, and suggest that stabilizing light environments and monitoring herd age structure could help reduce reproductive losses in commercial pig production.

## 1. Introduction

In pig production, reproductive efficiency is a key industry priority, and genetic programs increasingly aim to select females that maintain stable performance across challenging conditions. One important challenge is seasonal infertility, a multifactorial, largely non-infectious condition, associated with reduced sow reproductive performance; symptoms include anestrus, prolonged wean-to-estrus intervals, lower farrowing rates, and smaller litter sizes [1,2]. Its expression is influenced by parity/age, nutritional status, genetic background, and environmental conditions [3,4]. Within environmental conditions, increases in ambient temperature and overall heat load (HL), together with photoperiod shifts, are key triggers [5].

In production systems, maintaining gestating sows within the thermoneutral zone (18–20 °C) can significantly influence reproductive outcomes [6,7]. In this context, HL provides a more informative measure of risk, as it reflects the cumulative thermal challenge (driven by air temperature and modified by factors such as humidity and air movement) that determines the sow’s ability to dissipate heat [8]. When HL exceeds the thermoregulatory capacity, sows experience heat stress, triggering physiological responses that reduce uterine blood flow and disrupt endocrine function, thereby impairing fertilization and compromising embryonic development, particularly during early gestation [9,10,11]. Notably, these effects are not uniform across females and may vary by parity. Younger sows (parities 1–2) appear more vulnerable due to ongoing physiological maturation and competing energy demands for growth and lactation, whereas older sows (parity ≥ 3) tend to show greater resilience to thermal stress [12,13].

Moreover, photoperiod shifts are another critical factor to consider. Although domesticated pigs have been selected for continuous, year-round breeding, they still appear to retain some degree of photoperiod sensitivity inherited from their wild ancestors. In wild pigs, breeding is seasonally timed, with mating typically occurring in early winter and farrowing concentrated in late spring [14,15]. This retained sensitivity to day length may help explain reproductive declines during the summer–autumn transition, when photoperiod changes rapidly. Furthermore, the shifts in the timing of daylight increase nocturnal melatonin secretion, disrupt circadian rhythms, and impair hypothalamic–pituitary–ovarian signaling [16,17]. These effects may be more pronounced in primiparous sows, whose incomplete hypothalamic–pituitary–gonadal axis maturation results in weaker neuroendocrine responses to environmental cues [4,12,18].

Given these factors, the farm’s geographic location plays a central role in shaping the environmental stressors that must be managed. For example, in tropical regions, generally warm year-round with limited variation in day length [19], HL can remain elevated for extended periods, so reproductive pressure may be more closely linked to persistent thermal burden [20]. By contrast, subtropical regions, characterized by hot summers, milder winters, and greater seasonal variation in day length and temperature [21], often experience more pronounced seasonal fluctuations in HL and photoperiod, which may intensify reproductive disruption during specific windows (e.g., the summer–autumn transition) [22]. Consequently, in climates that are more extreme or variable, closer monitoring and control of environmental conditions is often needed to mitigate reproductive losses [23,24]. Therefore, targeted monitoring of in-barn conditions using automated sensors can support early detection of risk periods and timely mitigation strategies [23].

Farrowing success (FS) and litter size (LS) are known to vary with parity and environmental factors in commercial herds [2,5]. Therefore, this study aimed to identify in-barn HL, light variability (LV), and parity as farm-specific predictors of FS and LS in purebred Large White sows raised under subtropical and tropical conditions in Brazil. Prediction models were used to assess the individual and combined contributions of these factors across parities and climatic regions. It was hypothesized that higher HL and greater LV would negatively affect FS and LS, with differential predictive effects depending on parity and farm location. Understanding these relationships may improve knowledge of seasonal infertility and support farm-specific management strategies.

## 2. Materials and Methods

**Figure 1:**
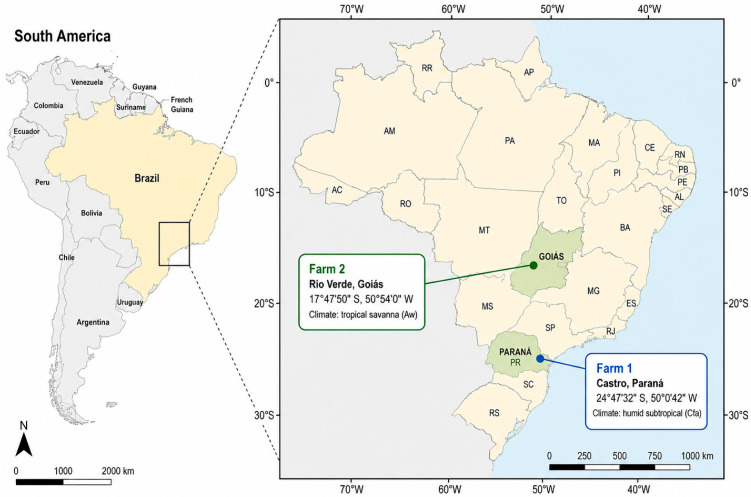
Geographic location of the study farms in Brazil. The left panel shows Brazil within South America, and the right panel highlights the two Brazilian states and farm locations included in the study.

Data were collected from two commercial Brazilian farms housing purebred Large White sows, located in distinct climatic regions, presented in Figure 1. Farm 1 was located in southern Brazil, in Castro, Paraná (24°47′32″ S, 50°0′42″ W), within a humid subtropical (Cfa) climate, while Farm 2 was in central-west Brazil, in Rio Verde, Goiás (17°47′50″ S, 50°54′0″ W), situated in a tropical savanna (Aw) climate, according to updated Köppen–Geiger climate classification maps [25].

### 2.1. Reproductive Performance

Reproductive data were obtained retrospectively from farm production records for the 2021 year (January–December 2021). On both farms, personnel recorded reproductive events manually as part of routine management, and these records were subsequently entered into a digital system; the database was then exported and checked for the analysis. The dataset included sow identification, parity, date of first insemination, date of a second insemination when required (i.e., re-service following return to estrus after the initial service), farrowing date, gestation length (days), farrowing outcome, LS (number of piglets born alive), and service sire. Farm 1 comprised 773 sows contributing 1523 service records in 2021, while Farm 2 comprised 1039 sows contributing 1957 service records across the same period. In both farms, sows were purebred Large White females, ranging from parity 1 to 8 in Farm 1 and parity 1 to 7 in Farm 2. Records were excluded when farrowing date, LS, HL, or LV data were missing, resulting in final analytical datasets of 1231 records from 719 sows for Farm 1 and 391 records from 391 sows for Farm 2 for the whole year.

Regarding reproductive health, all sows were vaccinated against major diseases (porcine parvovirus, erysipelas, and leptospirosis) [26] and no record of reproductive diseases were found for this year period on the farms. It is worth noting that Brazil is also recognized as a porcine reproductive and respiratory syndrome (PRRSV)-free country [27], and has the government implementing numerous disease contingency plans, including those addressing African Swine Fever [28].

### 2.2. Environmental Monitoring

Both farms used naturally ventilated housing with sidewall curtains as the only environmental control. Curtains were adjusted to regulate air exchange and solar heat gain (opened to increase airflow and cooling; closed to reduce drafts and limit heat loss), and no mechanical cooling/heating or artificial lighting control was used.

Environmental conditions were monitored using data loggers (HOBO MX1104—Onset Computer Corporation, Bourne, MA, USA) installed in March 2021 in the center of the gestation units at sow eye level (approx. 60–70 cm from the floor). The data loggers recorded ambient temperature and illuminance at 30 min intervals through December 2021. For each service event, in-barn maximum daily temperature was used to derive HL and illuminance data were used to calculate daily LV, as described below.

### 2.3. Study Design and Evaluation Period

FS and LS were analyzed for sows serviced between March and August 2021, the period typically associated with late summer–early autumn infertility in Brazil. Records followed by a subsequent insemination were classified as reinsemination records (RI) and used as a descriptive indicator of non-farrowing reproductive events. Because the retrospective farm-management database did not include clinical confirmation or diagnostic details, RI may include return to estrus, pregnancy loss, abortion, or other reproductive failure events. Therefore, RI records were retained for the characterization of FS but were not analyzed as a separate clinical outcome.

Based on an upper critical temperature of 25 °C [6,8], HL was used to quantify thermal stress. A plateau-linear function was applied: HL = 0 when the maximum daily temperature (MDT) was ≤25 °C, and HL = MDT − 25 when MDT > 25 °C, with negative values truncated to zero [12]. For simplicity, this parameter was hereafter referred to as “temperature”. As for photoperiod, under the barns’ natural lighting conditions, in-barn illuminance was recorded in lux (lx) using the data loggers. As no artificial lighting was used in the gestation units, illuminance reflected the daylight entering the barns and modulation caused by curtain management.

### 2.4. Statistical Analysis

To quantify LV, an expected illuminance profile, was defined (0 lx between 20:00 and 06:00; 240 lx between 06:00 and 20:00), adapted from Stuhlträger et al. [29]. Residuals were calculated as observed lx − expected lx, and daily variability was summarized as the root mean square error (RMSE) of residuals (RMSE = √[Σ(residual^2^)/n], where n is the number of observations per day). Higher RMSE values indicate larger and/or more frequent departures from the expected profile. HL and LV were standardized (z-scores) prior to modeling to aid comparability across farms and outcomes.

FS was defined as a binary outcome (1 = farrowed; 0 = did not farrow), where sows were classified as FS = 1 if not re-inseminated and had ≥1 piglet born alive, and FS = 0 if they had 0 piglets born alive, were re-inseminated, or had gestation length ≤ 108 days. It was analyzed using generalized linear mixed models with a binomial error distribution and logit link function (glmer function, lme4 package [30]). For each farm independently and for the combined dataset, the fixed effects included HL, LV, and parity (treated as a continuous covariate). For the combined model, farm was additionally included as a fixed effect. A random intercept per sow was included in all models to account for repeated service records per sow. For Farm 2, parity was dropped from the model due to rank deficiency, as all sows in this farm were primiparous (parity 1); therefore, parity effects could not be estimated. Effects are reported as odds ratios (ORs) with 95% confidence intervals (CIs), obtained by exponentiating the regression coefficients and their CI limits; OR > 1 indicates a positive association and OR < 1 indicates a negative association with farrowing success.

LS was analyzed using linear mixed models fitted with the lmer function from the lme4 package, with the same fixed effects structure as the FS models. For Farm 1 and the combined dataset, a random intercept per sow was included to account for repeated records. For Farm 2, as with the FS model, parity was not estimable given that all sows were primiparous, and no random intercept was included as each sow contributed a single record. Effects are reported as regression coefficients (β), representing the change in number of piglets born alive per unit increase in each standardized predictor; positive β values indicate an increase and negative β values indicate a decrease in litter size.

In addition to the regression models, a descriptive comparison restricted to primiparous sows was performed to facilitate interpretation of between-farm differences independent of parity range. For this comparison, primiparous records from Farm 1 were contrasted with Farm 2 during the study period. This comparison was used only for descriptive interpretation and was not intended to test farm or climate-zone effects statistically.

To explore exposure profiles, k-means clustering was performed on standardized values of HL, LV, and parity. Each sow contributed one observation, using their individual records directly. The number of clusters was determined using the Elbow method, in which the within-cluster sum of squares (WSS) is plotted against the number of clusters and the optimal k is identified at the point where additional clusters yield diminishing reductions in WSS (the ‘elbow’ of the curve) [31]. K-means was run with a fixed random seed (set.seed(123)) and 25 random starts. Clusters were visualized using principal component analysis (PCA) and described using group means for each variable.

All analyses were performed in R (R Core Team 2024; https://www.R-project.org, accessed on 3 June 2025) [32] using the packages lme4 [30], dplyr [33], factoextra [34], car [35] and sjPlot [36], all available through CRAN (https://cran.r-project.org, accessed on 3 June 2025). Statistical significance was assessed at *p* < 0.05.

## 3. Results

### 3.1. Reproductive Outcomes

Between March and August 2021, the final dataset comprised 732 service records from Farm 1 (601 unique sows, parities 1–8) and 233 service records from Farm 2 (233 unique sows, parity 1 only), as presented in Table 1. Overall FS was 89.3% in Farm 1 and 90.6% in Farm 2, while mean LS was 15.3 and 13.2 piglets, respectively. During the same period, RI represented 10.7% of service records in Farm 1 and 9.4% in Farm 2.

The results for FS and LS are presented separately, reflecting models evaluated both at the individual farm level and within the combined dataset.

Table 2 summarizes the logistic regression results for FS. In the combined analysis, parity (OR = 1.20; *p* = 0.003) and farm (OR = 1.72; *p* = 0.029) were the only significant predictors of FS, indicating higher odds of farrowing success with increasing parity and in Farm 2 compared with Farm 1. In farm-specific analyses, Farm 1 showed a significant positive association between parity and FS (OR = 1.20; *p* = 0.002), whereas temperature and LV were not significant predictors. In Farm 2, LV was negatively associated with FS (OR = 0.72; *p* = 0.028), while temperature was not significant.

Table 3 summarizes the results of the linear models for LS. In the combined analysis, parity was the strongest predictor of LS, showing a positive association with the number of piglets born alive (β = 0.47 piglets per parity increase; *p* < 0.001). Temperature also showed a small but significant positive association with LS (β = 0.28; *p* = 0.031). In farm-specific analyses, Farm 1 showed significant positive effects of both temperature (β = 0.36; *p* = 0.014) and parity (β = 0.47; *p* < 0.001) on LS, while LV showed a positive but non-significant trend. In Farm 2, LV was negatively associated with LS (β = −0.59; *p* = 0.020), whereas temperature was not significant.

When restricted to first-parity sows, Farm 2 showed numerically higher FS than the primiparous subset of Farm 1 (90.6% vs. 81.2%; *n* = 233 and *n* = 149, respectively), whereas mean LS was of similar magnitude between farms (13.22 vs. 13.98 piglets). Because this comparison was descriptive and not intended to test farm or climate-zone effects statistically, these values should be interpreted cautiously and may reflect herd structure, farm-level management, or other unmeasured factors in addition to climatic region.

### 3.2. Cluster Analysis

**Figure 2:**
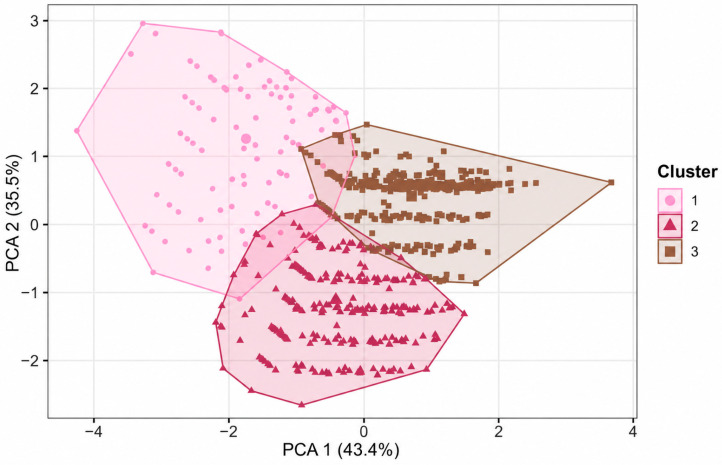
Cluster plot based on the principal component analysis of standardized heat load, light variability, and parity number, showing three distinct sow exposure profiles identified using k-means clustering.

*K*-means clustering identified three distinct sow exposure profiles based on standardized HL, LV, and parity. Cluster 1 comprised primarily intermediate-parity sows (parities 2–3) exposed to below-average HL and low LV; Cluster 2 consisted mainly of older sows (parity ≥ 4) under the lowest HL and LV values; and Cluster 3 was dominated by primiparous sows (parity 1) exposed to elevated HL and greater LV, indicating more variable and less favorable in-barn conditions. PCA supported this structure, with PC1 (43.4% of variance) driven primarily by environmental variables (HL and LV) and PC2 (35.5%) associated mainly with parity (Figure 2). Cluster distribution differed between farms: in Farm 1 sows were more evenly distributed across clusters, whereas in Farm 2 a higher proportion belonged to Cluster 3. These clusters describe co-occurring patterns of environmental exposure and parity rather than biological performance outcomes, providing complementary context to the regression analyses.

## 4. Discussion

Across the evaluated farms, parity consistently emerged as a major determinant of reproductive performance. In the combined model, each unit increase in parity was associated with 20% higher odds of FS (OR = 1.20; Table 2) and an increase of approximately 0.47 piglets born alive per litter (β = 0.47; Table 3), indicating that reproductive maturity and prior reproductive experience play a central role in determining sow performance during the late summer–early autumn period, exerting a stronger influence than environmental variation within the range observed [37]. These findings partially support the study hypothesis: while parity-dependent effects on reproductive outcomes were confirmed, the expected negative effects of higher HL and greater LV on FS and LS were not uniformly observed across farms, highlighting the farm-specific nature of environmental predictors as proposed in the study objective. This pattern is biologically plausible, as primiparous and low-parity sows are known to experience greater reproductive instability due to incomplete physiological maturity, competing demands for growth and lactation, and less stable endocrine regulation [2,18]. As parity increases within a moderate range, improvements in uterine capacity, ovarian function, and embryonic survival typically result in larger litters and a higher likelihood of maintaining pregnancy [38,39]. This framework may help explain differences between farms. Farm 2, consisting of predominantly primiparous sows, may not yet have reached peak reproductive performance, and improvements in FS are expected as these animals progress into intermediate parities [2,4]. In contrast, Farm 1 may include a higher proportion of sows that have already reached or passed peak reproductive capacity.

Interestingly, LS showed a small positive association with temperature. Although the detrimental impacts of heat stress on swine reproduction are well documented, this unexpected relationship likely reflects parity selection and confounding management factors rather than a direct biological benefit of increased temperatures [2,4]. The LS analysis is conditional on sows that successfully farrowed; under higher HL, more heat-sensitive females may be more likely to return to estrus or experience gestation loss, leaving a subset of more robust, high-performing sows contributing to observed litters [12]. In addition, HL is inherently correlated with season and management responses, such as enhanced cooling strategies, dietary adjustments, and increased supervision, which may partially offset thermal stress effects [7,40]. Together, these factors can reverse the apparent direction of association unless heat exposure is modeled by biologically sensitive reproductive windows and adequately controlled for seasonality and parity structure [3,41].

When farms were analyzed individually, distinct patterns emerged. In Farm 1 (subtropical region), parity was the strongest predictor of both FS and LS, with no significant associations between temperature or LV and FS. However, temperature showed a small but significant positive association with LS. The positive temperature effect on LS is unlikely to reflect a true biological benefit of heat and more likely represents seasonal confounding [12]. Services occurring during warmer months (June–August) took place after the documented late summer–early autumn infertility window (February–May), meaning these sows may have simply been serviced at a naturally more fertile time of year. Critically, temperature showed no effect on FS, the primary reproductive outcome, which would be expected if thermal conditions directly enhanced reproduction. This dissociation between temperature’s effect on LS but not FS suggests that the temperature signal is driven by timing rather than thermal physiology [41]. The lack of significant LV effects in Farm 1 is noteworthy given the region’s pronounced seasonal variation in photoperiod. This suggests that naturally driven photoperiod changes in subtropical environments, even during shorter autumn/winter days, may not substantially disrupt reproduction within the physiological tolerance ranges sows experience in this climate [42]. In contrast, we recall that parity emerged as a significant predictor of both FS (OR = 1.20) and LS (β = 0.47) in Farm 1, where sows ranged from parity 1 to 8, likely reflecting the well-documented progression from reproductive instability in early parities toward peak performance at intermediate parities [2]. These findings suggest that within Farm 1, physiological state was a stronger determinant of reproductive outcome than the environmental variation observed during the study period.

In Farm 2 (tropical region), temperature showed no significant association with either FS or LS, suggesting chronic exposure to high temperatures may limit the additional reproductive impact of within-year temperature variation [7]. In other words, sows acclimated to persistently high thermal conditions may experience limited additional stress from seasonal temperature fluctuations within their normal range. In contrast, LV showed a significant negative association with both FS and LS. Specifically, each unit increase in LV (RMSE z-score) was associated with 28% lower odds of FS (OR = 0.72; Table 2) and a reduction of approximately 0.59 piglets born alive per litter (β = −0.59; Table 3), indicating that greater day-to-day instability in barn illuminance meaningfully compromises both the likelihood of a successful pregnancy and the number of piglets produced. This is particularly relevant in tropical regions, characterized by relatively stable year-round photoperiod [2]. The observed effect of light variability, defined as day-to-day deviations in barn illuminance from the expected daylight profile, suggests that fluctuations in the in-barn environment, rather than absolute temperature or photoperiod duration, may represent a key stressor under these conditions [17]. Irregular light exposure may impair melatonin secretion patterns and disrupt hypothalamic–pituitary–gonadal axis signaling, compromising gonadotropin release and, consequently, ovarian function, embryo survival, and uterine receptivity [2,10]. Even when day length is consistent, variations in barn illuminance may interfere with sows’ perception of the light-dark cycle and circadian regulation [17]. Therefore, management strategies aimed at stabilizing light exposure, such as improved curtain handling to reduce daily illuminance fluctuations or the use of supplemental artificial lighting, may be more effective than thermal interventions alone for improving reproductive performance in tropical production systems [2,42].

The cluster analysis added key context for interpreting why the farms differed. Farm 1 showed a fairly even spread across the three clusters, indicating a herd with mixed parity and a range of environmental exposure profiles. In this more heterogeneous setting, variation in temperature and lighting did not meaningfully impair reproductive outcomes, suggesting that seasonal fluctuations in the subtropical environment stayed largely within physiological tolerance limits [3,42]. Any advantage expected from more stable lighting conditions (Clusters 1 and 2, characterized by low LV) was difficult to detect because even younger sows exposed to greater variability (Cluster 3) maintained adequate reproductive function, likely reflecting the farm’s milder environmental extremes and the timing of inseminations during naturally favorable reproductive periods [2]. By contrast, Farm 2 was dominated by Cluster 3, concentrating primiparous animals in the highest heat load and most variable lighting conditions. This herd structure is important for interpreting the between-farm results. In the descriptive comparison restricted to first-parity sows, Farm 2 showed numerically higher FS than the primiparous subset of Farm 1, whereas mean LS was of similar magnitude between farms. Because this comparison was not intended to test farm or climate-zone effects statistically, these values should be interpreted cautiously. Nevertheless, they suggest that the overall farm difference in FS observed in the combined model may not reflect a consistent advantage attributable to climate zone alone, but may also be influenced by herd structure, farm-level management, or other unmeasured factors [2,17].

The predominance of primiparous animals in Farm 2 also highlighted a structural vulnerability: the herd depended heavily on replacement females during the study period, and these physiologically immature sows were simultaneously exposed to high temperatures and pronounced day-to-day light instability within the barn. The negative association between LV and both farrowing success and litter size may therefore have emerged more clearly because it affected the largest and potentially most susceptible subgroup. In these animals, environmental instability and limited physiological maturity may act jointly to compromise reproductive performance [13]. The absence of older sows in Farm 2 also explains why parity effects could not be evaluated in the farm-specific analysis. Accordingly, differences in herd composition and monitoring periods should be considered when interpreting comparisons between farms.

Some additional aspects of the study design should be considered when interpreting the findings. Data were obtained from two commercial farms during a single production year and therefore primarily represent the climatic, management, and herd conditions evaluated. Studies involving additional farms and production years would help establish the consistency of these relationships across systems and over time. Barn dimensions were not available, and illuminance was monitored continuously at one representative fixed location within each barn. Although this approach enabled characterization of temporal changes in the light environment, the use of multiple sensors in future studies could provide a more detailed assessment of spatial variation in sow light exposure.

Within this context, the findings emphasize the importance of farm-specific, adaptive management, as the primary factors associated with reproductive performance were not uniform across production systems. From a practical standpoint, greater control over barn conditions, combined with real-time monitoring of temperature and light exposure, may help producers identify emerging patterns associated with seasonal infertility and intervene earlier [23,42]. In subtropical systems, attention to parity structure and breeding management may yield the greatest gains, whereas in tropical systems, stabilizing daily in-barn light conditions may represent an important management opportunity alongside thermal control [2,3]. Overall, integrating environmental monitoring with farm-specific interventions offers a feasible approach to supporting reproductive efficiency.

## 5. Conclusions

This study advances the understanding of seasonal infertility by demonstrating that the reproductive effects of in-barn HL and LV are context-dependent and strongly influenced by herd parity structure and climatic region. Rather than acting as uniform stressors, the evaluated environmental factors showed different predictive relevance across farms, with parity emerging as the main determinant of reproductive outcomes and LV representing an important risk factor in the tropical system, particularly in primiparous-dominated populations. These results indicate that integrating parity structure with environmental monitoring can improve the prediction of FS and LS, supporting the development of farm-specific early-warning tools for reproductive management. Additionally, future studies should validate these predictive factors across more farms, multiple years, and different genetic lines, while incorporating additional variables such as body condition score, feeding strategy, and detailed cooling and lighting management records to improve model accuracy and practical applicability.

## Figures and Tables

**Table 1:** Monthly number of service records, reproductive performance, and environmental indicators from Farm 1 and Farm 2 during March–August 2021.

Months	T (n)	MT (°C)	AD (h)	RI (%)	FS (%)	LS (n)
Farm 1 (*n* = 732 serviced records)						
3	95	33.6	12.2	10.5	89.4	15
4	117	33.3	11.5	9.4	90.6	15
5	135	32.9	10.9	10.3	89.6	15
6	121	32.0	10.6	9.0	90.9	15
7	119	28.3	10.8	8.4	91.6	15
8	145	32.6	11.3	15.1	84.8	14
Farm 2 (*n* = 233 serviced records)						
3	24	35.3	9.3	4.1	95.8	13
4	50	33.7	9.6	8.0	92.0	13
5	39	33.5	9.8	10.2	89.7	14
6	35	34.2	10.1	5.7	94.2	13
7	47	35.6	10.3	10.6	89.3	13
8	38	34.5	10.6	15.7	84.2	13

T = total number of service records (inseminations) per month; MT = monthly mean of the daily maximum in-barn temperature; AD = average daily hours per month with in-barn illuminance ≥ 240 lx between 06:00 and 20:00; RI = service records followed by a subsequent insemination, used as an indicator of non-farrowing reproductive events. Because this retrospective dataset did not include clinical confirmation or diagnostic details, RI may include return to estrus, pregnancy loss, abortion, or other reproductive failure events; FS = farrowing success, calculated as the percentage of service records resulting in farrowing; LS = mean litter size among successful farrowings.

**Table 2:** Logistic regression results for farrowing success based on environmental parameters and parity in combined and farm-specific models.

Variable	OR	95% CI	*p*-Value
Combined farms			
Temperature (HL, z-score)	1.095	0.920–1.304	0.305
Light variability (LV, z-score)	0.981	0.822–1.172	0.836
Parity	1.200	1.065–1.351	0.003
Farm (Farm 2 vs. Farm 1)	1.724	1.059–2.808	0.029
Farm 1			
Temperature (HL, z-score)	1.162	0.958–1.410	0.128
Light variability (LV, z-score)	1.093	0.878–1.362	0.427
Parity	1.201	1.067–1.351	0.002
Farm 2 *			
Temperature (HL, z-score)	0.834	0.530–1.310	0.431
Light variability (LV, z-score)	0.718	0.534–0.965	0.028

HL = heat load; LV = light variability; OR = odds ratio; CI = 95% confidence interval. Predictors HL and LV were standardized (z-scores). * Farm 2 comprised parity-1 services only, so parity was not evaluated within this farm.

**Table 3:** Linear regression results for litter size based on environmental parameters and parity in combined and farm-specific models.

Variable	Estimate (β)	95% CI	*p*-Value
Combined farms			
Temperature (HL, z-score)	0.281	0.026–0.536	0.031
Light variability (LV, z-score)	0.047	−0.217–0.311	0.728
Parity	0.467	0.311–0.624	<0.001
Farm (Farm 2 vs. Farm 1)	−0.440	−1.132–0.253	0.213
Farm 1			
Temperature (HL, z-score)	0.355	0.071–0.639	0.014
Light variability (LV, z-score)	0.255	−0.035–0.546	0.085
Parity	0.471	0.314–0.627	<0.001
Farm 2 *			
Temperature (HL, z-score)	0.020	−0.580–0.620	0.948
Light variability (LV, z-score)	−0.591	−1.086–−0.097	0.020

HL = heat load; LV = light variability; β = regression coefficient (change in piglets per unit increase in predictor); CI = 95% confidence interval. HL and LV were standardized (z-scores). * Farm 2 comprised parity-1 services only, so parity could not be evaluated within this farm.

## Data Availability

Due to commercial confidentiality, the raw data are not publicly available.

## Abbreviations

AD, average daily hours with in-barn illuminance ≥ 240 lx; AIC, Akaike information criterion; Aw, tropical savanna climate; CI, confidence intervals; Cfa, humid subtropical climate; FS, farrowing success; HL, heat load; LS, litter size; LV, light variability; lx, lux; MDT, maximum daily temperature; MT, monthly mean of the daily maximum in-barn temperature; OR, odds ratio; PCA, principal component analysis; RI, reinsemination records; RMSE, root mean square error; T, temperature; and WSS, within-cluster sum of squares.
